# Postnatal Depression among Rural Women in South India: Do Socio-Demographic, Obstetric and Pregnancy Outcome Have a Role to Play?

**DOI:** 10.1371/journal.pone.0122079

**Published:** 2015-04-07

**Authors:** Siddharudha Shivalli, Nandihal Gururaj

**Affiliations:** Department of Community Medicine, Yenepoya Medical College, Yenepoya University, Deralakatte, Mangalore, Karnataka, India; Institute of Psychiatry, UNITED KINGDOM

## Abstract

**Introduction:**

Postnatal depression (PND) is one of the most common psychopathology and is considered as a serious public health issue because of its devastating effects on mother, family, and infant or the child.

**Objective:**

To elicit socio-demographic, obstetric and pregnancy outcome predictors of Postnatal Depression (PND) among rural postnatal women in Karnataka state, India.

**Design:**

Hospital based analytical cross sectional study

**Setting:**

A rural tertiary care hospital of Mandya District, Karnataka state, India.

**Sample:**

PND prevalence based estimated sample of 102 women who came for postnatal follow up from 4th to 10th week of lactation.

**Method:**

Study participants were interviewed using validated kannada version of Edinburgh Postnatal Depression Scale (EPDS). Cut-off score of ≥13 was used as high risk of PND. The percentage of women at risk of PND was estimated, and differences according to socio-demographic, obstetric and pregnancy outcome were described. Logistic regression was applied to identify the independent predictors of PND risk.

**Main Outcome Measures:**

Prevalence, Odds ratio (OR) and adjusted (adj) OR of PND

**Results:**

Prevalence of PND was 31.4% (95% CI 22.7–41.4%). PND showed significant (P<0.05) association with joint family, working women, non-farmer husbands, poverty, female baby and pregnancy complications or known medical illness. In binomial logistic regression poverty (adjOR: 11.95, 95% CI:1.36–105), birth of female baby (adjOR: 3.6, 95% CI:1.26–10.23) and pregnancy complications or known medical illness (adjOR: 17.4, 95% CI:2.5–121.2) remained as independent predictors of PND.

**Conclusion:**

Risk of PND among rural postnatal women was high (31.4%). Birth of female baby, poverty and complications in pregnancy or known medical illness could predict the high risk of PND. PND screening should be an integral part of postnatal care. Capacity building of grass root level workers and feasibility trials for screening PND by them are needed.

## Introduction

Pregnancy and postpartum are considered as high risk periods for the emergence of psychiatric disorders. Postnatal depression (PND) is one of the most common psychopathology in these phases. PND describes non-psychotic depressive episodes, with loss of interest, insomnia, and loss of energy experienced by mothers within the period of 4 to 6 weeks after delivery [1]. A wide range of PND prevalence (10–42%) has been reported across the globe [2–11]. It is considered a serious public health issue because of its devastating effects on mothers, families, and infants or children [12]. Accurate estimates of PND prevalence are difficult to obtain as cultural norms may affect women’s reporting of their symptoms and methods used to determine prevalence rates impact their accuracy [13,14].

Available evidence suggests that Asian women have higher rates of depression during pregnancy than North American women [15]. PND seems to have multi factorial etiology encompassing demographic, economic, psychosocial, obstetric and medical risk factors. Cross cultural common PND risk factors have been pointed out in resource-rich and resource-limited settings [16]. However, unique factors such as birth of baby girl, protective influence of traditional rituals, financial insecurity, marital violence, lack of social support have emerged as risk factors for PND in low and medium income countries like India, Pakistan and Turkey [6,8,17].

PND and its risk factors have been explored by many authors in Indian setting [5,6,7,8], however, no research is conducted so far in local study setting. In view of paucity of research among rural mothers in local setting and to aid the early diagnosis of PND, this study aimed to elicit socio-demographic, obstetric and pregnancy outcome predictors of PND among postnatal women in rural part of Mandya district, Karnataka state, India.

## Methods

### Study setting

This study was conducted in outpatient section of Department of Obstetrics and Gynecology of a tertiary care hospital situated in rural part of Mandya district, Karnataka state, India from June to Nov 2012. The studied hospital is attached to a rural medical college recognized by Medical Council of India with teaching facility for both under and post graduates in obstetrics and gynecology. It caters to the need of nearby villages and *talukas* and linked with many government health programmes for the benefit of rural poor.

The total population of Mandya district is 1.92 million. Situated in southern part of Karnataka state, it is a prominent agricultural district blessed with the irrigation waters of river Cauvery and Hemavathi [18]. More than half of the population is involved in agriculture. Most of the industries that are run in the district depend on agriculture for their raw material. Sugar Mills, Jaggery making units, Rice Mills are the prominent industries of this district [18].

### Study design and sample

Cross sectional study design was adopted to accomplish the study objective. All the women who came for postnatal follow up to studied hospital from June to July 2012 formed the sampling frame. We decided to study at least 96 postnatal mothers based on minimum estimated PND prevalence of 10% with 6% precision, 95% confidence level [19]. All the women who came for follow up from 4^th^ to 10^th^ week of postnatal period from June to Nov 2012 were included. Women with acute severe illness or cognitive impairment or not willing to consent for voluntary participation were excluded. In a day maximum of two postnatal women, satisfying inclusion and exclusion criteria were selected randomly from the outpatient list of Department of Obstetrics and Gynecology.

### Study tools

A pretested semi-structured interview schedule was used to elicit information pertaining to socio-demographic profile, obstetric and pregnancy outcome. Anonymity of the study participants was maintained to enhance the participation rate and also to ensure confidentiality.

Following details were sought after; socio-demographic profile: age, religion, education, working status, economic status, husband’s occupation and family type; obstetric details: duration of married life, parity, any complications in the last pregnancy and known medical or psychiatric disease; pregnancy outcome: gender of the baby, mode and type of delivery.

Woman’s literacy status was classified as literate (if she can read and write with understanding in any language) or illiterate (can neither read nor write or can read but cannot write in any language) and literacy level was the highest level of education completed. (Census India 2011) [20].

Working status of the woman was categorized as employed (engaged in economically productive work) or unemployed. Similarly, the family structure was categorized as nuclear (consisting of a couple and/or their dependent children) or joint (a number of married couples and their children where all men are related by blood, who live under one roof, eat from common kitchen, hold property in common, and participate in common family worship) [21]. And the type of delivery was stratified as preterm (live birth before 37 completed weeks of gestation) or term (live birth between 37 and 42 weeks of gestation) delivery [22].

In India, public distribution system issues ration card to families by assessing their economic status. In this study the type of ration card possessed by the woman was taken as proxy indicator of her economic status i.e. yellow and blue colored cards for below and above poverty line families, respectively.

Edinburgh postnatal depression scale (EPDS): It is a 10 item self report scale based on 1 week recall, designed to screen PND in community [23]. Each item is rated from 0 to 3, yielding a total score of 0–30. Seven of its items are reverse-scored. In India, the Kannada (local language) version of EPDS has been validated to detect antenatal depression, and found to have a sensitivity of 100% and specificity of 84.9%, at a cut-off score of ≥13 [24]. For this study, a cut-off score of ≥13 on Kannada version of EPDS was used to diagnose PND. It was pre tested by the second author on 10 postnatal mothers under the guidance of a qualified psychiatrist to ensure that questions were easily understood by mothers and responses were correctly interpreted by the author. Psychiatrist approved the pilot study and rest of the interviews were conducted by second author only to avoid inter observer bias. Woman with EPDS score of ≥13 was referred to a psychiatrist for further evaluation.

#### Statistical analysis

Data was analyzed using Statistical Package for the Social Sciences (SPSS) for Windows, Version 16.0. Chicago, SPSS Inc. Results were expressed as frequencies and proportions for categorical variables and mean and standard deviations for continuous variables. The prevalence of PND was calculated as percentage of women who scored ≥ 13 in EPDS. Chi-squared test was applied to capture the differences in proportions of PND across socio-demographic, obstetric and pregnancy outcome variables. Fischer’s exact p was considered if more than 20% of the cells had an expected count of less than 5.Crude odds ratios (OR) with 95% confidence intervals were calculated for all the study variables. Binomial logistic regression was run to compute adjusted odds ratios (adjOR) for variables with significant association (p<0.05) in univariate analysis. Model fit was assessed using the Hosmer-Lemenshow test.

### Ethical approval

Institution review board and ethics committee of Adichunchanagiri Institute of Medical Sciences, Balagangadharanatha Nagara, Mandya District, Karnataka, India, approved the study protocol. Informed written consent was taken from all the respondents for voluntary participation in local language (*kannada*).

## Results

We approached 118 women who came for postnatal follow up during the study period and satisfied inclusion and exclusion criteria. However, only 102 of them consented for voluntary participation and non-participation rate was 13.6%.

Study participants’ mean age was 23.1 ± 2.9 years (range: 18–33 years) and mean duration of married life was 2.66 ± 2.4 years (range: 1–11 years). All the postnatal women were living with their partner. Most of them were Hindus (88.2%) by religion. [Table 1] Joint family system was in vogue among them (63.7%). Literacy rate was 61.8% and three fourths of them were economically below poverty line. Unemployed women (73.5%) and primiparas (67.6%) formed the majority among the study participants. Most of the husbands (98.7%) of housewives were farmers by occupation. Almost all of the primiparas (53.9% of the total) had delivered within one year of marriage.

**Table 1:** Key socio-demographic, obstetric and pregnancy outcome features of the study participants.

**Study variable**	**N**	**%**
**Age (years)**		
18–20	18	17.6
21–25	64	62.7
26–30	17	16.7
>30	3	2.9
**Religion**		
Hindu	90	88.2
Muslim	12	11.8
**Type of family**		
Joint	65	63.7
Nuclear	37	36.3
**Education status**		
Illiterate	39	38.2
Literate	63	61.8
**Economic status**		
Above poverty line	23	22.5
Below poverty line	79	77.5
**Working status of postnatal woman**		
Unemployed	75	73.5
Employed	27	26.5
**Husband’s occupation**		
Farmer	75	74.5
Others	25	25.5
**Parity**		
1	69	67.6
2	26	25.5
3	7	6.9
**Type of delivery**		
Full term	99	97.1
Pre term	3	2.9
**Mode of delivery**		
Normal	84	82.4
Cesarean section	18	17.6
**Gender of the baby**		
Female	41	40.2
Male	61	59.8
**Complications in pregnancy/Known medical illness**		
Yes	10	9.8
No	92	90.2
**Total**	102	100

As many as 13.7% (n = 14) of them had delivered by cesarean section. Child sex ratio was 672 girls per 1000 boys. Almost every tenth postnatal woman had one or the other complication or medical illness in the last pregnancy. Preeclampsia, eclampsia, anemia and polycystic ovarian syndrome were some of them. None of the studied mothers had abortion or still birth in the last pregnancy.

The EPDS score of 32 study participants was ≥13 and the prevalence of PND was 31.4% (95% CI: 22.7–41.4%). Univariate analysis revealed significant (p<0.05) association of socio-demographic (type of family, economic status, working status and husband’s occupation), obstetric (complications in pregnancy/known medical illness) and pregnancy outcome (gender of the baby) variables with PND. [Table 2]

**Table 2:** Association of socio-demographic, obstetric and pregnancy outcome variables with risk of postnatal depression (n = 102).

	EPDS score					
Study variable	≥ 13		< 13		Total	p
	N	%	N	%		
**Duration of married life**						
< 1 year	16	29.1	39	70.9	55	0.591
≥1 year	16	34	31	66	47	
**Age**						
≤21 years	13	36.1	23	63.9	36	0.446
>21 years	19	28.8	47	71.2	66	
**Education**						
Literate	20	31.7	43	68.3	63	0.918
Illiterate	12	30.8	27	69.2	39	
**Religion**						
Muslim	3	25	9	75	12	0.749^††^
Hindu	29	32.2	61	67.8	90	
**Type of family**						
Joint	25	38.5	40	61.5	65	0.041^†^
Nuclear	7	18.9	30	81.1	37	
**Working status**						
Employed	13	48.1	14	51.9	27	0.028^†^
Unemployed	19	25.3	56	74.7	75	
**Husband’s occupation**						
Farmer	19	25.3	56	74.7	75	0.028^†^
Others	13	48.1	14	51.9	27	
**Socio-economic status**						
Below poverty line	31	39.2	48	60.8	79	0.002^†^
Above poverty line	1	4.3	22	95.7	23	
**Parity**						
Multipara	14	42.4	19	57.6	33	0.096
Primipara	18	26.1	51	73.9	69	
**Pregnancy planning**						
Planned	20	36.4	35	63.6	55	0.249
Unplanned	12	25.5	35	74.5	47	
**Mode of delivery**						
Normal	26	31	58	69	84	0.476
Cesarean section	6	33.3	12	66.7	18	
**Type of delivery**						
Pre term	2	66.7	1	33.3	3	0.231^††^
Full term	30	30.3	69	69.7	99	
**Gender of the newborn**						
Female	19	46.3	22	53.7	41	0.008^†^
Male	13	21.3	48	78.7	61	
**Complications in pregnancy/Known medical illness**						
Yes	8	80	2	20	10	0.001^†^ ^††^
No	24	26.1	68	73.9	92	

^†^significant;

^††^Fisher’s exact test

Risk of PND showed significant association with woman from joint family (OR: 2.73, 95% CI: 1.02–7.0), employed women (OR:2.73, 95% CI:1.1–6.8), husbands who were non-farmers (OR: 2.73, 95% CI: 1.1–6.8), poverty (OR:14.2, 95% CI: 1.8–110.8), birth of female baby (OR:3.19, 95%CI: 1.34–7.6) and occurrence of complications in pregnancy or known medical illness (OR:11.3, 95% CI:2.27–57.1). [Table 3]

**Table 3:** Crude and adjusted Odds ratios for the risk of postnatal depression according socio-demographic, obstetric and pregnancy outcome variables (n = 102).

Study variable	Crude Odds ratio	95% CI	P	Adjusted^†^	95% CI	p
				Odds ratio		
**Duration of married life**						
< 1 year	0.794	0.34–1.8	0.591	-	-	-
≥1 year	1					
**Age**						
≤21 years	1.4	0.59–3.3	0.446	-	-	-
>21 years	1					
**Education**						
Literate	1.05	0.44–2.5	0.918	-	-	-
Illiterate	1					
**Religion**						
Muslim	0.701	0.17–2.8	0.749	-	-	-
Hindu	1					
**Type of family**						
Joint	2.73	1.02–7.0	0.041^¶^	2.8	0.85–8.97	0.092
Nuclear	1			1		
**Working status**						
Employed	2.73	1.1–6.8	0.028^¶^	2.8	0.16–50.7	0.486
Unemployed	1			1		
**Husband’s occupation**						
Others	2.73	1.1–6.8	0.028^¶^	1.016	0.06–18.1	0.991
Farmer	1					
**Socio-economic status**						
BPL^‡^	14.2	1.8–110.8	0.002^¶^	11.95	1.36–105.0	0.025^¶^
APL^§^	1			1		
**Parity**						
Multipara	2.09	0.87–5.0	0.096	1.658	0.519–5.3	0.394
Primipara	1			1		
**Pregnancy planning**						
Unplanned	0.6	0.26–1.4		-	-	-
Planned	1					
**Mode of delivery**						
Cesarean section	0.97	0.30–2.64	0.401	-	-	-
Normal	1					
**Type of delivery**						
Pre term	4.6	0.4–52.7	0.231	-	-	-
Full term	1					
**Gender of the newborn**						
Female	3.19	1.34–7.6	0.008^¶^	3.6	1.26–10.23	0.017^¶^
Male	1			1		
**Complications in pregnancy/Known medical illness**						
Yes	11.3	2.25–57.1	0.001^¶^	17.4	2.5–121.2	0.004^¶^
No	1			1		

Cox and Snell R Square = 0.365 & Nagelkerke R Square = 0.487

^†^By binomial logistic regression;

^‡^Below poverty line;

^§^Above poverty line;

^¶^Significant (p<0.05)

In binomial logistic regression poverty (adjOR: 11.95, 95% CI: 1.36–105), birth of female baby (adjOR: 3.6, 95% CI: 1.26–10.23) and complications in pregnancy or known medical illness (adjOR: 17.4, 95% CI: 2.5–121.2) remained as independent predictors of PND. [Table 3]

## Discussion

### Main findings

Substantial proportion (31.4%) of lactating women who came for follow up, from 4 to 10 weeks of post-partum, to our study setting is at risk of PND. It is associated with poverty, birth of baby girl and occurrence of complications in pregnancy or known medical illness.

### Interpretation

Pregnancy and childbirth are unique life events and cannot be reduced to primarily biological events since the social and cultural contexts are central to the subjective and collective experiences of women. [25] Women’s age, literacy, social economic status, religion and culture do have a significant influence on pregnancy experiences and her mental health. The present study was an attempt to explore the aforesaid and biological risk factors of PND.

Reported prevalence of PND in this study was 31.4% (95% CI 22.7–41.4%) and is quite high. Other hospital and community based studies in India have reported lower prevalence of PND ranging from 11% to 23% [5,6,7,8,26]. Very wide range of PND prevalence has been reported in studies from China (11%), United Arab Emirates (15.8%), Zimbabwe (16%), Brazil (20.7%), South Africa (34.7%), and Pakistan (40%) [27,28,29,30]. The present study may have overestimated the actual PND prevalence as EPDS is a screening tool but not confirmatory. Other reasons for variations in PND prevalence could be attributed to study tool and technique differences, socio-cultural norms and poverty levels.

Poverty was an independent predictor of PND in our study. Poverty lead financial problems may act as an additional stressor, especially at the juncture where another family member is being added [26]. This is corroborated by the reports of studies from low and middle income countries [7, 16, 31, 32]. However, such association was not seen in other studies [11,26,30].

Although, type of family, woman’s and husband’s occupations showed significant association with PND in univariate analysis but binominal logistic regression ruled out their effect. Was it a serendipitous finding or these women had other protective influences, e.g. better social-support and marital quality etc, needs further investigation [26]. Some studies [8,33] have found an association of PND with age and working status of the mother.

Present study did not show significant association between PND and planning of pregnancy, mode and type of delivery. Similar findings are reported by Hegde S et al [26] and Milgrom J et al [34]. On the contrary, unplanned and unwanted pregnancies, operative procedures and difficult labor have been reported as risk factors for PND in previous studies [16,27]

In India preference to male child is deeply entrenched. In Indian culture, girl goes to her husband’s house, changes her last name to husband’s last name and expected to continue his family lineage. Whereas male stays with parents till death and he will continue their family pedigree, support parents in their old age, conduct funeral rites and inherit ancestral property. Birth of baby girl is considered as a family and social stressor in Indian societies and hence could be a strong predictor of PND [26]. The present study and other Indian studies from Karnataka [26], Goa [7] and Tamilnadu [8] corroborate this gender bias.

Occurrence of complications or medical illness during pregnancy was also the predictors of PND in this study. However, none of the mother reported any psychiatric disease during pregnancy or in the past. This could be due to lack of knowledge or cultural norms affecting womens’ reporting of their symptoms [13,14]. Various studies have reported depression during pregnancy is an important predictor of PND [7,8,9].

There is agreement and disparity in the prevalence and various predictors of PND across India and the globe. However, it is strongly recommended that PND screening should be an integral part of postnatal care owing to simplicity of screening by EPDS and to avert devastating effects on mother and child.

In most of the rural India, postnatal care is being rendered by Auxiliary Nurse Midwife (ANM) for every 5,000 rural populations [35]. Community health volunteers called Accredited Social Health Activists (ASHAs), for every 1000 rural populations, have been engaged under the National Rural Health Mission to facilitate the ANM to render maternal and child health services and establish a link between community and the healthcare system [36]. Feasibility trials for screening PND among rural Indian women by these grass root level workers are needed.

#### Conclusion

Risk of PND among rural postnatal women was high (31.4%). Birth of female baby, poverty and complications in pregnancy or known medical illness could predict the high risk of PND. PND screening should be an integral part of postnatal care. Capacity building of grass root level workers and feasibility trials for screening PND by them are needed.

### Limitations

Unfortunately women with abortion or stillbirth did not come for follow up during the study period. Therefore we could not study their association with PND. We also did not consider other factors such as quality of social support, domestic violence, gender preference, women empowerment etc. EPDS is a screening tool for PND and not confirmatory. In rural parts of Karnataka state 64.7% of the women receive postnatal care within two weeks of delivery [37] and relatively a small sample was studied in our study. Therefore, results would apply to women attending hospitals/clinics and cannot comment on those who do not receive postnatal care. Nonetheless, the present study gives valuable hints to practicing obstetricians and primary care physicians in resource constrained rural India for early identification and apt preventive intervention for women at risk of PND.

### Details of ethics approval

Institution review board of Adichunchanagiri Institute of Medical Sciences, Balagangadharanatha Nagara-571448, Karnataka, India approved the study protocol in April 2012.
